# ATI-2307 Exhibits Equivalent Antifungal Activity in *Cryptococcus neoformans* Clinical Isolates With High and Low Fluconazole IC_50_

**DOI:** 10.3389/fcimb.2021.695240

**Published:** 2021-06-23

**Authors:** Elliot S. Gerlach, Sophie Altamirano, J. Marina Yoder, Tony S. Luggya, Andrew Akampurira, David B. Meya, David R. Boulware, Joshua Rhein, Kirsten Nielsen

**Affiliations:** ^1^ Department of Microbiology and Immunology, University of Minnesota, Minneapolis, MN, United States; ^2^ Infectious Diseases Institute, College of Health Sciences, Makerere University, Kampala, Uganda; ^3^ Division of Infectious Diseases and International Medicine, Department of Medicine, University of Minnesota, Minneapolis, MN, United States

**Keywords:** ATI-2307, *Cryptococcus*, azole, antifungal, susceptibility, resistance, IC_50_, T-2307

## Abstract

Half maximal inhibitory concentrations (IC_50_) to the experimental drug ATI-2307 and complete inhibition (IC_90_) of the common clinically used antifungal drug amphotericin B were determined by microbroth dilution assay for a collection of 69 clinical isolates of *Cryptococcus neoformans* from Uganda that had high fluconazole IC_50_ values. The majority of the clinical isolates tested had fluconazole IC_50_ at or above 8 µg/mL, but were susceptible to both amphotericin B (IC_90_ ≤1 μg/mL) and ATI-2307 (IC50 ≤0.0312 µg/mL). No correlation between increased fluconazole minimum inhibitory concentration (MIC) and ATI-2307 or amphotericin B MIC was observed, suggesting that the cellular changes impacting fluconazole susceptibility did not impact the effectiveness of ATI-2307. Our results suggest that ATI-2307 is a promising new antifungal drug for use in the context of high fluconazole or other antifungal drug MICs and/or in combination drug therapy regimens.

## Introduction

*Cryptococcus neoformans* is an environmentally acquired pathogenic yeast that causes the disease cryptococcal meningitis. Disease is typically observed in immunocompromised individuals, particularly those with advanced HIV/AIDS, and thus is a leading cause of mortality in sub-Saharan Africa (Park et al., 2009; Rajasingham et al., 2017; Hurtado et al., 2019). For cryptococcal meningitis, the small repertoire of antifungal drugs remains a critical limitation. The location of the infection in the central nervous system, where the blood-brain barrier complicates or limits drug dissemination, further reduces the antifungal drugs that can be used successfully for cryptococcal meningitis therapy (Felton et al., 2014; Roemer and Krysan, 2014). In addition, because many cryptococcal meningitis patients are immunocompromised and thus often receiving other drug treatments, drug-drug interactions must be considered (Vadlapatla et al., 2014). The most effective antifungal drug for treatment of cryptococcal meningitis, amphotericin B, is known for its potential to cause significant side effects and need for intravenous administration (Klepser, 2011).

There is increasing documentation of differences in antifungal drug MICs in *C. neoformans* isolates from cryptococcal meningitis patients (Pfaller et al., 1998; Assing et al., 2003; Archibald et al., 2004; Mdodo et al., 2011). These differences are best documented in the azole drug fluconazole where IC_50_ values range from 0.25 to >256 µg/mL (Pfaller et al., 2011; Chen et al., 2015; Smith et al., 2015; Bongomin et al., 2018; Chesdachai et al., 2019; Naicker et al., 2020). To date, clinical breakpoints (CBPs) are not clearly established within the field. Studies to identify epidemiologic cut-off values show IC_50_ of 4 or 8 µg/mL, but there is significant variability depending upon geographic region and across time (Mdodo et al., 2011; Pfaller et al., 2011; Smith et al., 2015; Pharkjaksu et al., 2020). Thus, clinical drug susceptibility and resistance are challenging to define in *C. neoformans* and instead strains are often classified based on high or low IC_50_/IC_90_ values upon *in vitro* MIC assay.

Fluconazole is a broad-spectrum antifungal commonly used for the treatment of other fungal infections observed in immunocompromised patients, such as mucosal candidiasis and fungal skin infections, and is commonly prescribed to HIV-seropositive patients. This previous fluconazole exposure for other indications may be causing the fluctuations in fluconazole MIC observed in cryptococcal meningitis patients (Naicker et al., 2020). Alternatively, azole fungicides are also commonly used in agriculture, and azole cross-resistance is well documented in other fungal species (Verweij et al., 2009). Given that *C. neoformans* is environmentally acquired, this agricultural azole use could also be driving changes in fluconazole MIC of clinical strains (Smith et al., 2015).

Fluconazole can be used in combination therapy with amphotericin B and is the drug of choice for consolidation therapy after initial amphotericin B treatment (Molloy et al., 2018). Currently, fluconazole still has a major role in prevention of meningitis in the preemptive treatment of persons with early disseminated cryptococcal infection, termed cryptococcal antigenemia (Meya et al., 2010; Nalintya et al., 2018). Fluconazole targets the ergosterol biosynthesis pathway, disrupting fungal cell membrane structure and formation. Given the increasing recognition of fluconazole resistance within *C. neoformans* clinical isolates, development of additional drug treatments, with different modes of action, is needed.

ATI-2307 (formerly T-2307 at FUJIFILM Toyama Chemical Co. Ltd) is a pentamidine-like compound with antifungal activity currently under development at Appili Therapeutics, Inc. ATI-2307 has a broad spectrum of activity against many fungal pathogens including *Candida* spp, *Aspergillus* spp., and *C. neoformans*. The mode of action of ATI-2307 is different from the azoles; ATI-2307 acts *via* selectively inhibiting yeast mitochondrial respiratory chain complexes III and IV (Mitsuyama et al., 2008; Shibata et al., 2012; Yamashita et al., 2019). Here we tested the antifungal activity of ATI-2307 on isolates of *C. neoformans* with high fluconazole IC_50_ sampled from cryptococcal meningitis patients enrolled in multiple clinical trials in Uganda. This analysis was performed to demonstrate that ATI-2307 maintains activity in a diverse set of *C. neoformans* isolates common to the patient population for which the drug may be developed, and to determine if there was a correlation between elevated fluconazole MIC and increased ATI-2307 MIC.

## Materials and Methods

### Drugs and Dilutions

4-{3-[1-(3-{4-[amino(imino)-methyl]phenoxy}propyl)piperidin-4-yl]propoxy}benzamidine (T-2307, ATI-2307) was provided by Appili Therapeutics, Inc. (Nova Scotia, Canada) as a trihydrochloride pentahydrate salt. Fluconazole and amphotericin B were purchased from Sigma-Aldrich (St. Louis, MO). A 10 mg/mL stock solution of ATI-2307 was prepared in sterile water then further diluted to a working stock solution of 59.904 µg/mL free base ATI-2307. Final microbroth dilution assay concentrations were determined from a value of 68.7% free base to salt, with the molecular weight of each being 437.59g/mol and 637.04g/mol, respectively. A 50 mg/mL stock solution of fluconazole was prepared in DMSO. Amphotericin B was acquired pre-diluted to 250 µg/mL in sterile water. The ATI-2307 test concentrations ranged from 0.0004875 – 0.2496 µg/mL, the fluconazole test concentrations ranged from 0.25-128 µg/mL, and the amphotericin B test concentrations ranged from 0.0078125 – 4 µg/mL.

### Inoculum Preparation

Clinical isolates of *C. neoformans* with high, intermediate, and low levels of fluconazole susceptibility, obtained as part of the ASTRO trials (Rhein et al., 2016; Rhein et al., 2019) or COAT trial (Boulware et al., 2014) were plated onto yeast-peptone-dextrose (YPD) plates containing 0.04 mg/mL chloramphenicol and incubated at 30°C for 48 hours. Overnight cultures were subsequently prepared in YPD broth containing 10 µg/mL chloramphenicol and incubated at 30°C with shaking. Cells were centrifuged and washed 3 times with sterile water, resuspended, and a 1:100 dilution was prepared for quantification *via* hemocytometer. The final inoculum of each isolate for the microbroth dilution MIC assay was prepared to the subsequent EUCAST specifications (Arendrup et al., 2017) in sterile water.

### Microbroth Dilution MIC Assays

Broth microdilution assays were carried out according to the EUCAST protocol following the subsection “*Cryptococcus* spp” recommendations, using a 2% glucose RPMI-1640 medium (Sigma R8755) with a final inoculum concentration of 0.5 x 10^5^ – 2.5 x 10^5^ (Arendrup et al., 2017). The RPMI-1640 used contained no phenol red indicator. Immediately following inoculation, optical density at 600nm wavelength (OD600) measurements were obtained on a Biotek Synergy H1 Hybrid reader (Winooski, VT). Plates were then incubated 72 hours at 37°C, and a second OD600 measured. The IC_50_ or IC_90_ for each strain was determined based on analysis of the well turbidity measurements using the OD600, as described in Smith et al. (2015) with IC_50_ defined as the drug concentration at which growth was limited to 50 percent of baseline growth in the absence of drug and IC_90_ defined as the drug concentration at which growth was limited to 10 percent of baseline growth (calibrated as no visible growth) in the absence of drug. KN99α (Nielsen et al., 2003), with a known fluconazole IC_50_ of 2 µg/mL and amphotericin B IC_90_ of 0.5 µg/mL (Smith et al., 2015) and ATI-2307 IC_50_ of 0.0078 µg/mL (this study), was included as an inter-assay calibration reference in every MIC plate to verify accuracy across MIC plates.

### Analysis

Association between fluconazole, amphotericin B, and ATI-2307 susceptibility was compared with generalized linear regression models using GraphPad Prism version 9.0.2 (San Diego, CA).

## Results

**Figure 1:**
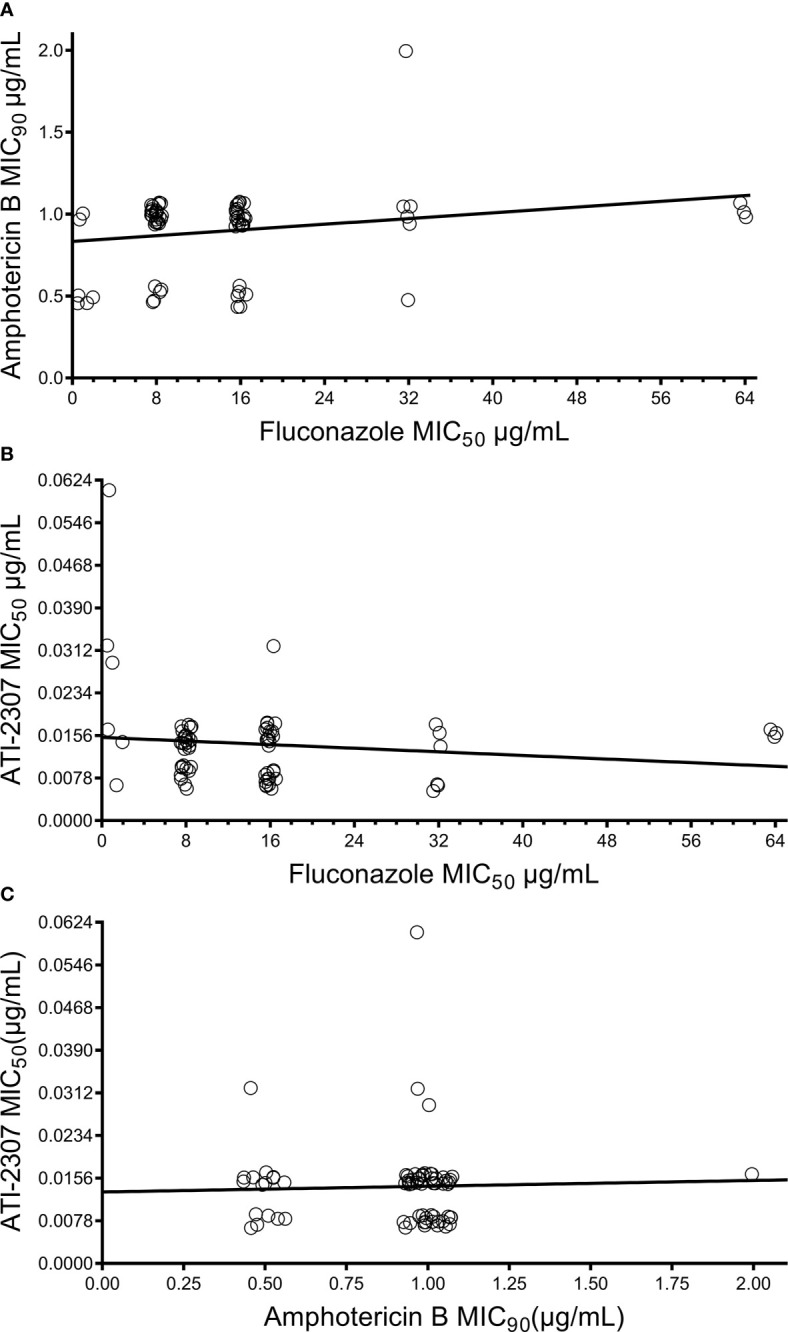
Correlation between fluconazole IC_50_, amphotericin B IC_90_, and ATI-2307 IC_50_ values for *Cryptococcus neoformans* clinical isolates. No correlation was observed between **(A)** fluconazole and amphotericin B susceptibilities (rho = 0.2049, *P* = 0.21), **(B)** fluconazole and ATI-2307 susceptibilities (rho = -0.1690, *P* = 0.1650), or **(C)** amphotericin B and ATI-2307 susceptibilities (rho = 0.0125, *P* = 0.9190).

We analyzed 69 C*. neoformans* clinical isolates from Uganda with varying levels of fluconazole IC_50_ from <8 µg/mL (n=6), 8 µg/mL (n=28), 16 µg/mL (n=22), and 32 or 64 µg/mL (n=9) (**Table 1**). All 68 isolates had low amphotericin B IC_90_ of 0.5 µg/mL (n=16), 1.0 µg/mL (n=52), and 2.0 µg/mL (n=1). Similar to previous reports (Casadevall et al., 1993; Perea and Patterson, 2002), we observed no correlation between fluconazole IC_50_ and amphotericin B IC_90_ (**Figure 1A**, rho=0.2049, *P*=0.0912). All 69 isolates also fell within the previously established ATI-2307 IC_50_ range of 0.0078 – 0.0624 µg/mL (Mitsuyama et al., 2008) for *C. neoformans* strains (**Table 1**). Again, we observed no correlation between fluconazole IC_50_ and ATI-2307 IC_50_ (**Figure 1B**, rho=-0.1690, *P*=0.1650). In addition, we showed no correlation between amphotericin B IC_90_ and ATI-2307 IC_50_ values (**Figure 1C**, rho=0.0125, *P*=0.9190). Finally, we determined MIC_50_ and MIC_90_ values for ATI-2307 and amphotericin B, respectively, and showed they remain equivalent when the strains were analyzed based on fluconazole IC_50_ values (**Table 2**).

**Table 1:** Fluconazole and ATI-2307 IC_50_, and amphotericin B IC_90_ values, for high IC_50_ fluconazole *Cryptococcal neoformans* clinical isolates*.

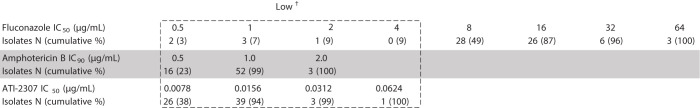

IC_50_ and IC_90_ values were determined using the EUCAST microbroth dilution method (Arendrup et al., 2017).

*A total of 69 isolates were screened, and data are presented as the number (cumulative percentage) of isolates with growth inhibition at (or below, for cumulative percentage) the indicated drug concentration.

^†^Low IC_50_/IC_90_ ranges are indicated in the dashed box. Fluconazole IC_50_ values < 8 μg/mL are considered low. IC_90_ values ≤1 µg/mL are considered low for amphotericin B. IC_50_ values ≤0.0624 µg/mL are susceptible to ATI-2307 based on murine studies (Mitsuyama et al., 2008).

**Table 2:** MIC_50_ and MIC_90_ for ATI-2307 and amphotericin B are equivalent across *Cryptococcus neoformans* clinical isolates with differing fluconazole IC_50_.

	Fluconazole IC_50_ (µg/mL)				
	Total (n = 69)	IC_50_ ≤ 2 (n = 6)	IC_50_ = 8 (n = 28)	IC_50_ = 16 (n = 26)	IC_50_ ≥ 32 (n = 9)
**ATI-2307**					
**IC_50_ min**	0.0078	0.0078	0.0078	0.0078	0.0078
**IC_50_ max**	0.0624	0.0624	0.0156	0.0312	0.0156
MIC_50_*	0.0156	0.0156	0.0156	0.0078	0.0156
MIC_90_*	0.0156	0.0624	0.0156	0.0156	0.0156
**Amphotericin B**					
**IC_90_ min**	0.5	0.5	0.5	0.5	0.5
** IC_90_ max**	2.0	1.0	1.0	1.0	2.0
MIC_50_*	0.5	0.5	0.5	0.5	0.5
MIC_90_*	1.0	0.5	1.0	1.0	1.0

*MIC_50_ and MIC_90_ are the drug concentrations at which 50% or 90% of the strains tested have an IC_50_ or IC_90_ at or below the indicated drug concentration, respectively.

## Discussion

The *in vitro* EUCAST broth microdilution assay IC_50_ measurements for *C. neoformans* clinical isolates showed no correlation between high fluconazole IC_50_ and activity of the experimental antifungal drug ATI-2307 or amphotericin B. All the Ugandan clinical isolates tested had IC_50_ values for ATI-2307 that were below the 0.0624 µg/mL threshold previously identified in *C. neoformans* by Mitsuyama et al. (2008), irrespective of fluconazole sensitivity. While we only analyzed strains from Uganda and differences in fluconazole resistance are observed in populations from across the globe, given the completely different mode of action of ATI-2307 it is likely that our results showing no association between ATI-2301 and fluconazole resistance will be representative for *C. neoformans*. Consistent with the *in vitro* activity, similar drug concentrations are effective in murine models of *C. neoformans* (Mitsuyama et al., 2008) and *C. gattii* (Nishikawa et al., 2017), suggesting that therapeutic levels of ATI-2307 against *Cryptococcus* species can be achieved *in vivo*.

A similar study with the novel azole-derivative VT-1129 showed a positive correlation with increasing fluconazole IC_50_, although the increase in VT-1129 IC_50_ was not biologically significant due to the enhanced potency of VT-1129 compared to fluconazole (Nielsen et al., 2017). In contrast, and as expected due to the different modes of action of ATI-2307 and fluconazole, our study shows no reduction in ATI-2307 potency in the high fluconazole IC_50_ clinical isolates. As such, ATI-2307 may be a good candidate to pursue for frontline therapy in areas where high fluconazole MICs are known to occur, or as second line therapy when drug treatment has failed due to fluconazole resistance.

High fluconazole MICs have been documented in several countries throughout the world (Chen et al., 2015; Smith et al., 2015; Naicker et al., 2020). Furthermore, a systematic review of fluconazole MICs by Chesdachai and colleagues shows the median MIC_50_ is trending upwards, with the potential for current fluconazole dosing guidelines proving inadequate for cryptococcosis in the context of these rising MICs (Chesdachai et al., 2019). While Smith et al. (2015) found no effect of higher fluconazole IC_50_ in the context of HIV patients receiving combination therapy with amphotericin B 800 mg/day consolidation therapy in Uganda, a similar study by Nasri and colleagues (2016) with both transplant recipients and individuals with HIV found that patients with high fluconazole IC_50_ values that received voriconazole or higher-dose fluconazole (≥800 mg) for consolidation therapy were more likely to survive. These results highlight the effectiveness of utilizing drugs with different modes of action in combination therapy approaches to drug treatment, but also the need for new non-azole drugs that can be used during consolidation therapy.

In conclusion, our studies show that ATI-2307 is fully active against clinical isolates of *C. neoformans* with high fluconazole IC_50_ values. This novel drug has strong potential as a new antifungal therapeutic to increase the arsenal of drugs with differing modes of action that can be explored for use, either in combination or monotherapy approaches, for the treatment of cryptococcal meningitis or antigen positive cryptococcosis.

## Data Availability Statement

The original contributions presented in the study are included in the article. Further inquiries can be directed to the corresponding author.

## Author Contributions

EG, SA, JMY, DB and KN contributed to conception and design of the study. TL, AA, JR, DM, and DB collected the clinical isolates, designed, and performed the clinical trials, EG performed the statistical analysis. EG and KN wrote the first draft of the manuscript. All authors contributed to the article and approved the submitted version.

## Funding

This study was supported by Appili Therapeutics, Inc. research award CON000000083519. The funder was not involved in the study design, collection, analysis, interpretation of data, the writing of this article or the decision to submit it for publication. Additional funding to support the collection and analysis of the clinical isolates was supported by NIH grants U01AI089244 and R01NS086312 to DB and R01NS118538 to KN.

## Conflict of Interest

KN received funding from Appili Therapeutics Inc.

The remaining authors declare that the research was conducted in the absence of any commercial or financial relationships that could be construed as a potential conflict of interest.
